# High prolactin levels in dihydropteridine reductase deficiency: A sign of therapy failure or additional pathology?

**DOI:** 10.1002/jmd2.12236

**Published:** 2021-06-29

**Authors:** Nicola Vitturi, Livia Lenzini, Concetta Luisi, Miryam Carecchio, Giorgia Gugelmo, Francesco Francini‐Pesenti, Angelo Avogaro

**Affiliations:** ^1^ Division of Metabolic Diseases, Department of Medicine‐DIMED, University Hospital University of Padova Padova Italy; ^2^ Emergency Medicine Unit, Department of Medicine‐DIMED, University Hospital University of Padova Padova Italy; ^3^ Department of Neuroscience University of Padova Padova Italy; ^4^ Division of Clinical Nutrition, Department of Medicine‐DIMED, University Hospital University of Padova Padova Italy

**Keywords:** BH_4_, DHPR, dopamine, prolactin

## Abstract

We report the case of a 22‐year‐old man with a diagnosis of dihydropteridine reductase (DHPR) deficiency who progressively developed movement disorders and epilepsy. Despite L‐Dopa supplementation the patient continued to show high prolactin levels, with a discrepancy between the neurological clinical picture and the hormonal biochemical levels. For this reason, other potential causes were ruled out by performing a cerebral magnetic resonance imaging, which demonstrated a solid lesion in the pituitary gland strongly suggestive of a prolactinoma. As the association between metabolic disorders affecting biogenic amine synthesis and prolactinoma has not been previously reported in humans, this report suggests that a critical evaluation of the use of prolactin as a guide for therapy dosage should be made in patients with DHPR deficiency disorders.

## 1 INTRODUCTION

Dihydropteridine reductase (DHPR) deficiency is the second most frequent autosomal recessive disorder of tetrahydrobiopterin (BH_4_) synthesis. It is characterized by an elevation of phenylalanine (Phe) and low levels of biogenic amines.^1^, ^2^ The clinical presentation may be an asymptomatic elevation of Phe on newborn screening or a progressive deterioration of neurological picture despite low Phe diet.^3^

The aim of treatment is to control the Phe levels and to correct the deficiency of biogenic amines in the central nervous system.

Phe levels are controlled by low Phe diet life long, while biogenic amine replacement therapy is given as l‐Dopa and 5‐hydroxytryptophan (5‐HT). Due to side effects, l‐dopa and 5‐HT should be started in a low dose and increased gradually.

Monitoring cerebrospinal fluid (CSF) concentration of homovanillic acid (HVA) as a marker of dopamine turnover and 5‐hydroxyindoleacetic acid (5‐HIAA) for serotonin metabolism is not feasible in clinical practice due to the need of repeating lumbar puncture, which is an invasive procedure. Hence, prolactin plasma level may be useful in monitoring l‐dopa deficiency due to its reverse feedback with l‐dopa synthesis.^4^

Treatment with folinic acid is also needed to prevent folate deficiency.

## 2 CASE REPORT

A 22‐year‐old man was initially diagnosed with phenylketonuria (PKU) during newborn metabolic screening; after 2 months, due to the onset of sialorrhea more complete metabolic evaluation was conducted, including biochemical screening (high biopterin levels in urine, very low 5‐HIAA, HVA and folate, low MHPG [3,4‐dihydroxyphenylglycol], and very high biopterin levels in CSF), enzymatic assay (no DHPR activity detectable in peripheral blood cells erythrocytes and leukocytes), and genetic analysis (homozygous variant in exon 1 of *QDPR* gene (c.13_32 dup; p. V13fs); hence, a diagnosis of variant hyperphenylalaninemia due to DHPR deficiency^5^ was made, and he was started on nutritional and pharmacological therapy.

His development was normal until the age of 3 when he had a generalized tonic‐clonic seizure, and carbamazepine was started. Few months later, he developed focal motor status epilepticus of left frontotemporal origin that became refractory and required treatment with several antiseizure drugs and anesthetics. Cerebral magnetic resonance imaging (MRI) performed at that stage showed multiple bilateral subcortical white matter lesions consistent with hypoxic‐ischemic lesions; a possible cardiac arrest as a trigger of the status epilepticus was hypothesized, but never documented on clinical or instrumental grounds. He recovered from mechanical ventilation after 2 months, but he was left with motor aphasia, right hemiparesis, and cognitive impairment.

During the follow‐up, he showed plasma Phe and the ratio of Phe/tyrosine within the normal range. Despite this metabolic stability and ongoing antiseizure treatment, he progressively developed drug‐resistant epilepsy, with daily focal to bilateral tonic‐clonic seizures, tonic seizures, and electroencephalography (EEG) findings, suggesting epileptic encephalopathy. Clinically, he showed a spastic tetraparesis, with superimposed dystonic movements of upper and lower limbs as well as cervical dystonia.

His treatment consisted in antiseizure drugs (clobazam, topiramate, levetiracetam, perampanel, sodium valproate), hydroxytryptophan, folinic acid, l‐dopa (melevodopa/carbidopa 100/250 mg three times a day).

Patient was referred to our Adult Hereditary Metabolic Clinic at the age of 20: he was wheelchair bound, alert but unable to communicate verbally, could perform some simple motor commands, and showed no rest tremor but diffuse muscular rigidity, so that l‐dopa daily dose was progressively increased. We also determined plasma prolactin levels, as suggested from literature: prolactin, physiologically, is inversely related to dopamine levels, so it can be considered a marker to guide the doses of dopamine replacement therapy, rather than using more invasive approaches (ie, biogenic amines levels in cerebrospinal fluid).

**FIGURE 1:**
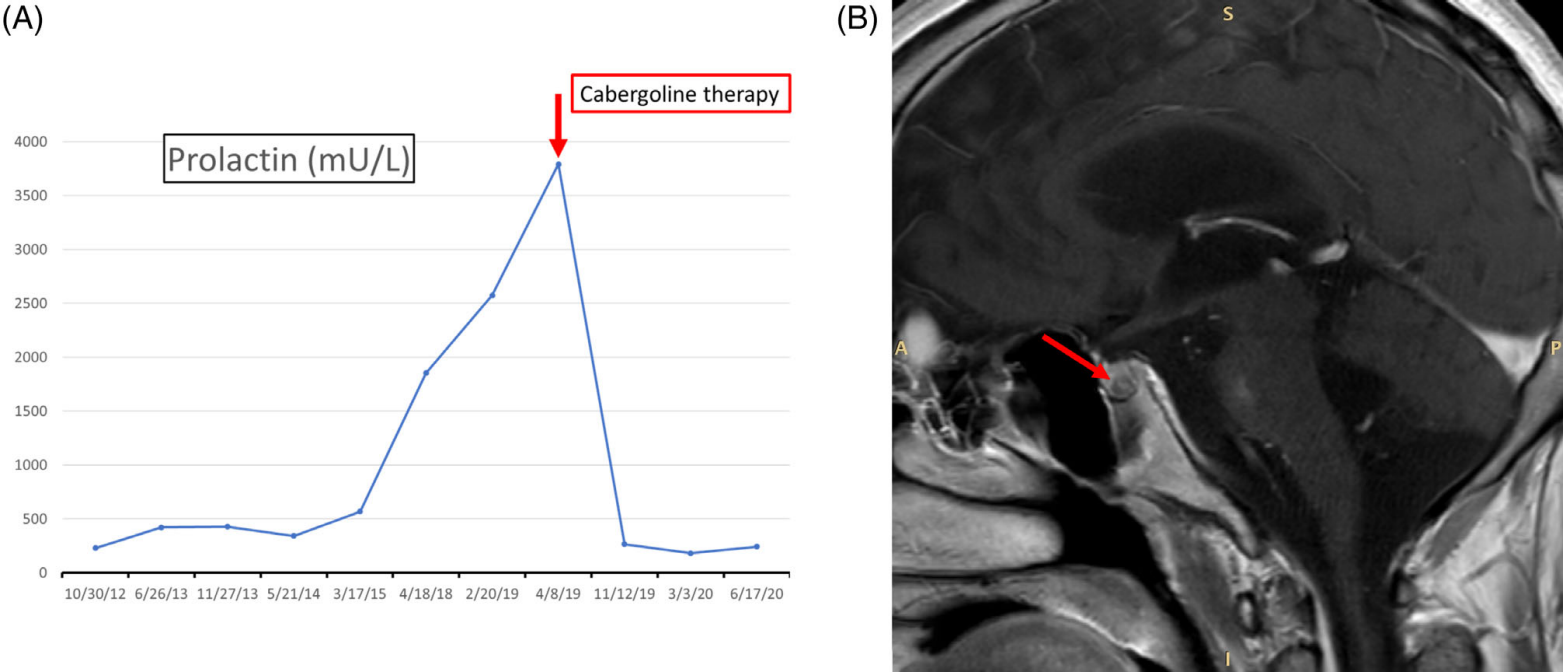
A, Prolactin levels (*Y*‐axis, mU/L) during the patient's follow‐up (*X*‐axis: blood samples date). B, Magnetic resonance imaging of brain and pituitary gland; red arrow shows in the left sector of the pituitary gland a hypointense signal area of 5 mm diameter, determining a slight salience of the pituitary profile, consistent with a small pituitary adenoma

Prolactin plasmatic level measurements at different times are reported in Figure 1A.

Following l‐dopa dosage adjustment (up to melevodopa/carbidopa 100/25 mg five times a day), the clinical picture showed a patterned stiffening and hyperextension of the upper right limb, sometimes involving the lower right limb or contralateral one, lasting about 20 seconds. Sweating and redness in the face were also present, while sphincteric release or tongue biting were not observed. The frequency of these episodes, which were consistent with dystonic posturing, was higher at night (up to 20 per night). All these symptoms were unmodified despite benzodiazepine administration. The frequency of epileptic seizures remained unchanged. The patient underwent long‐term monitoring video‐EEG to better evaluate these episodes and cerebral MRI (Figure 1B). The EEG did not show electric correlates of the dystonic posturing episodes. Despite a clinical picture consistent with dystonic posturing, possibly due to an excessive l‐dopa administration (as sometimes observed in peak dose dystonia/dyskinesia in Parkinson's disease, namely in the context of a neurodegenerative disease), prolactin levels did not decrease as expected after adequate l‐dopa administration.

The MRI showed the presence of bihemispheric large malacic lesions in the subcortical white matter, mostly in the semi‐oval centers and fronto‐parietal subcortical regions, more on the left side. Other bilateral cerebellar ischemic lesions were evident. As compared to previous MRI scan, signs of cortical atrophy were more pronounced and a moderate enlargement of ventricles was also present. In the left sector of the pituitary gland, a hypointense 5 mm area determining a slight salience of the pituitary profile was shown, being consistent with a pituitary adenoma. We therefore hypothesized that the patient's hyperprolactinemia was caused by a previously undiagnosed prolactinoma and not by l‐dopa deficiency. Moreover, the detection of cerebral bilateral hemispheric ischemic lesions explained the presence of tetraparesis.

The patient was then treated with cabergoline (0.5 mg ½ cp twice a week) and l‐dopa was reduced to 400 mg a day. The new treatment progressively reduced dystonic movements. After 4 months, prolactin levels returned within normal limits. At the last evaluation, the patient showed a good clinical picture, with a reduction of neurological signs.

## 3 DISCUSSION

**FIGURE 2:**
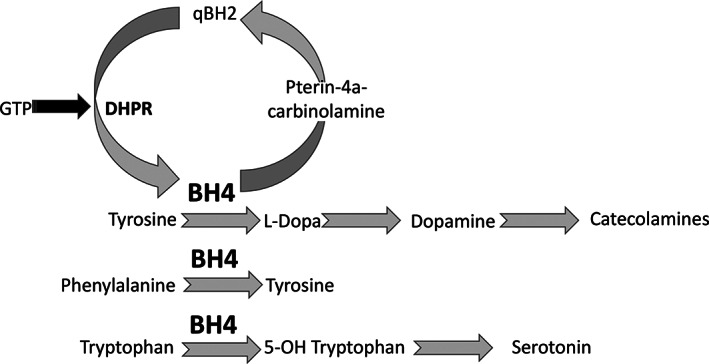
BH_4_ metabolism and function. GTP, guanosine triphosphate; BH_4_, tetrahydrobiopterin; qBH2, dihydrobiopterin (quinone); DHPR, dihydropteridine reductase

DHPR deficiency (OMIM#261630) is a rare recessive metabolic disease caused by biallelic mutations in the *QDPR* gene, leading to a deficient q‐dihydropteridine reductase activity: this causes an absolute reduction in tetrahydrobiopterin (BH_4_) levels,^3^ and an impairment of phenylalanine to tyrosine conversion but, unlike the most frequent PKU types, this condition leads also to a deficient synthesis of biogenic amines from tyrosine and tryptophan (Figure 2).

Sapropterin dihydrochloride (an analogue of BH) therapy is controversial in DHPR deficiency (unlike others BH_4_ deficit syndromes) due to a potential increased in 7,8‐dihydrobiopterin (BH_2_) production and a decreased BH_4_/BH_2_ ratio resulting in a worsening of clinical symptoms; however, guidelines^3^ suggest its use also in DHPR deficiency since the evidences against are scarce. The goal of therapy is to normalize biogenic amine levels by administering hydroxy‐tryptophan and l‐dopa (the direct dopamine precursor), while restricting dietary phenylalanine intake (to avoid its toxic accumulation in the brain). The effect of l‐dopa supplementation may be monitored clinically or biochemically by determining biogenic amine levels in the CSF. The need for a noninvasive marker has led to hypothesize the use of plasma prolactin levels; in fact, its strong, negative correlation to dopamine levels may be used for both diagnosis and follow‐up.^4^

In our patient, the development of movement disorders (dystonic movements usually related to dopamine excess in the context of neurodegenerative diseases such as Parkinson's disease) seemed not to correlate with high prolactin levels (that are considered a marker of dopamine deficiency). The discrepancy between the clinical picture and the presence of an inappropriately elevated prolactin level suggested the need for a deeper investigation on the possible causes of the latter condition. Indeed, the MRI demonstrated the presence of a pituitary prolactinoma.

The association between metabolic disorders affecting biogenic amine synthesis and prolactinoma has not been previously reported in humans affected by BH_4_ metabolism disorders, while in mice,^6^ the chronic inactivation of dopamine secretion causes an enlargement of pituitary gland due to hyperplastic hypertrophic lactotrophs and multifocal prolactinomas.

Despite the lack of a human model, we could hypothesize that the association described in our patient may reflect the same pathological pathway described by Hnasko and colleagues: a possible chronic endogenous dopamine deficiency that leads to a lack in D2R simulation may augment the risk of oncogenic mutation and promote pituitary tumor growth. Interestingly, in animal models, estrogen secretion is needed to promote the development of prolactinoma, while our male patient did not show pathological estrogen levels.

The possible association between DHPR deficiency and prolactinoma poses a clinical challenge in managing BH_4_‐deficiency disorders. The role of prolactin as a marker of dopamine deficiency, and its use as a guide for therapy dosages should be critically revised as pointed by the present clinical case, not only at the time of disease diagnosis but also during follow‐up. Variations in prolactin levels inconsistent with patient's clinical picture or not responsive to l‐dopa dosage modifications must be carefully monitored and evaluated to rule out underlying potentially curable endocrine conditions.

## 4 CONCLUSIONS

The present clinical case suggests that the measurement of prolactin as a guide for therapy dosages in the treatment of BH_4_ deficiency disorders should be critically reconsidered when a metabolic disorder of biogenic amine synthesis and prolactinoma coexist.

## CONFLICT OF INTEREST

The authors declare that they have no conflict of interest.

## AUTHOR CONTRIBUTIONS

Nicola Vitturi made metabolic evaluation of the patient and wrote the text; Livia Lenzini and Angelo Avogaro revised the manuscript; Concetta Luisi and Miryam Carecchio made the neurological evaluation of the patient; Giorgia Gugelmo and Francesco Francini‐Pesenti followed the nutritional care of the patient.
